# Inequalities in breast cancer incidence and mortality in women with and without disabilities in South Korea: A population-based cohort study

**DOI:** 10.1016/j.pmedr.2025.103242

**Published:** 2025-09-12

**Authors:** Hee-Yeon Kang, Eunjung Park, Thi Tra Bui, Byungmi Kim, Jin-Kyoung Oh

**Affiliations:** a Department of Public Health & AI, National Cancer Center Graduate School of Cancer Science and Policy, 323 Ilsan-ro, Ilsandong-gu, Goyang-si, Gyeonggi-do 10408, Republic of Korea; b Division of Cancer Prevention, National Cancer Control Institute, National Cancer Center, 323 Ilsan-ro, Ilsandong-gu, Goyang-si, Gyeonggi-do 10408, Republic of Korea

**Keywords:** Breast cancer, Disability, Incidence, Mortality, Health inequalities

## Abstract

**Objective:**

Women with disabilities often face functional limitations and comorbidities that increase cancer risk. This study investigated breast cancer incidence and mortality among women with and without disabilities in South Korea.

**Methods:**

We analyzed 2.87 million cancer-free women aged ≥30 who participated in the 2002–2003 national health screening program, linking the National Health Insurance Database, Cancer Registry, and cause-of-death records (2003–2019). Standardized incidence ratios (SIRs), standardized mortality ratios (SMRs), and mortality-to-incidence (M/I) ratios were calculated by disability status. Cox proportional hazards models with piecewise follow-up estimated adjusted hazard ratios (aHRs) for breast cancer outcomes.

**Results:**

Among 296,689 women with disabilities, 3579 developed breast cancer and 465 died from it; among 2.58 million without disabilities, 46,267 developed it and 3779 died. Women with disabilities had lower incidence (SIR ratio 0.86, 95 % CI 0.83, 0.90) but comparable breast cancer-specific mortality (SMR ratio 1.00, 95 % CI 0.89, 1.13). Cox models showed consistently lower aHRs for breast cancer incidence and mortality early on, but aHRs for all-cause mortality increased over time.

**Conclusions:**

Despite lower incidence, women with disabilities experienced similar breast cancer-specific mortality and higher long-term all-cause mortality. These findings underscore persistent inequalities and highlight the need for timely, equitable cancer care.

## Highlights

• Women with disabilities had lower breast cancer incidence but comparable mortality.
• They faced inequalities in screening access, diagnostic timeliness, and cancer care.
• Systemic barriers may underlie these inequalities in breast cancer outcomes.

## 1 Introduction

In 2021, approximately 1.3 billion people—16 % of the global population—were living with disabilities (World Health Organization, 2022). Prevalence increases with age, affecting one in three adults over 60, with women at higher risk (World Health Organization, 2011, World Health Organization, 2022). As populations age, the number of older individuals with disabilities is expected to rise, increasing the burden of chronic disease and cancer (Iezzoni, 2022). People with disabilities experience worse health outcomes than those without, including higher rates of functional limitations and chronic illness, shorter life expectancy, and increased mortality (Bahk et al., 2022; Kuper and Heydt, 2019; Majer et al., 2011; World Health Organization, 2022). They also face adverse social determinants of health, such as poor living conditions, low socioeconomic position, cultural barriers, and limited access to healthcare (Iezzoni, 2022; World Health Organization, 2011).

Among these inequalities, breast cancer presents a particular challenge for women with disabilities. Breast cancer is the most frequently diagnosed cancer and the leading cause of cancer-related death among women worldwide, accounting for 24.5 % of cases and 15.5 % of cancer deaths in 2020 (Sung et al., 2021). In South Korea (hereafter “Korea”), breast cancer incidence and mortality have continued to rise, despite downward trends in other major cancers (Kang et al., 2023).

The National Cancer Screening Program offers free or low-cost biennial mammography, yet access barriers persist for disadvantaged groups, including people with disabilities (Kang, 2022). Despite advances in screening and treatment, inequalities in breast cancer outcomes remain (Andiwijaya et al., 2022; Gross et al., 2020; Iezzoni et al., 2021; Keegan et al., 2023). Reduced access to timely diagnosis and treatment likely contributes to worse outcomes among women with disabilities.

Previous Korean studies have examined breast cancer inequalities by income, region, and education (Choi et al., 2018; Park et al., 2023; Shin et al., 2020). However, few have assessed long-term trends in cancer incidence and mortality using registry-linked data.

This study aimed to investigate inequalities in breast cancer incidence and mortality among women with and without disabilities in Korea between 2003 and 2019, using nationwide population-based data.

## 2 Methods

### 2.1 Data sources

We used the National Health Information Database (NHID; 2002–2019), a nationwide administrative database managed by the National Health Insurance Service of Korea (approval No. NHIS-2022-1-785). The NHID covers more than 50 million Koreans under a universal, single-payer healthcare system and includes sociodemographic information, healthcare utilization data, national health screening results, and mortality records (Seong et al., 2017).

The NHID is widely used in longitudinal epidemiologic research and enables linkage to external data sources via unique personal identifiers. We linked it to the Korea Central Cancer Registry (1999–2019), which provides nationwide cancer statistics with 98.2 % completeness as of 2021 (Park et al., 2024; Shin et al., 2005), and to cause-of-death records from Statistics Korea (2002–2019).

### 2.2 Study design and participants

**Fig. 1:**
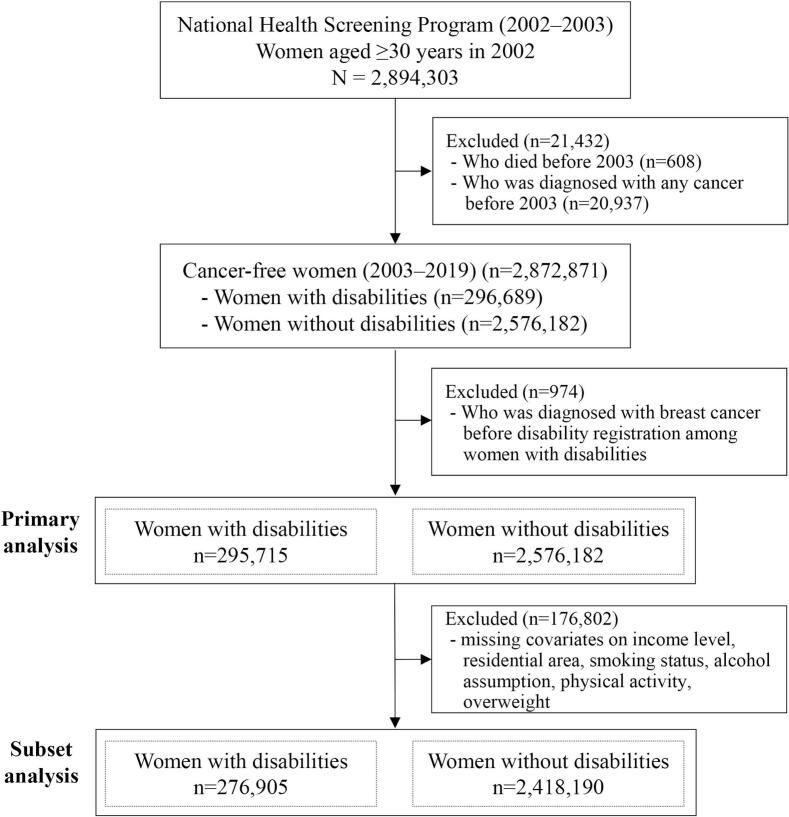
Flow chart of study participant selection in the National Health Screening Program (2002−2003).

We conducted a retrospective cohort study of women aged ≥30 years who participated in the general health screening program between January 1, 2002, and December 31, 2003. Of 14,005,088 eligible women, 2,894,303 (20.7 %) were screened during this period. After excluding 21,432 women with prior cancer diagnoses (*n* = 20,937) or who died before 2003 (*n* = 608), a total of 2,872,871 participants were included in the final analysis (Fig. 1).

### 2.3 Main exposure and outcomes

The main exposure, disability status, was identified from the Korea National Disability Registration System (2002–2019), which is integrated into the NHID. Established in 1988, this system requires medical certification by specialists under the Act on Welfare of Persons with Disabilities (Jeon et al., 2015; Kim et al., 2023).

The primary outcome, incident breast cancer, was defined as the first diagnosis of primary invasive breast cancer (ICD-O-3 site code C50, behavior code /3) between January 1, 2003, and December 31, 2019, according to Korea Central Cancer Registry records. Carcinoma in situ (behavior code /2 or ICD-10 code D05) was excluded. Follow-up began on January 1, 2003, and continued until breast cancer diagnosis, death, or December 31, 2019, whichever occurred first, regardless of other cancer diagnoses. Breast cancer-specific mortality was defined as death with an underlying cause of breast cancer (ICD-10 C50) in Statistics Korea records.

### 2.4 Covariates

Covariates included age group (10-year intervals); income level (quintiles based on insurance premiums); residential area (metropolitan, urban, rural); smoking status (current or not); alcohol use (≥1/week or rarely); physical activity (≥1/week or none); overweight status (BMI ≥25 kg/m^2^); Charlson Comorbidity Index (CCI: 0, 1–2, ≥3); and breast cancer screening history (ever or never). All variables were derived from the 2002–2003 general health screening questionnaires in the NHID, prioritizing 2002 data. CCI was calculated using insurance claims data (Charlson et al., 1987; Quan et al., 2005). Screening history was based on National Cancer Screening Program records prior to diagnosis for cases and across the full follow-up period (2002–2019) for non-cases, reflecting baseline age-based eligibility.

### 2.5 Statistical analysis

We calculated age- and calendar year-adjusted standardized incidence ratios (SIRs) and standardized mortality ratios (SMRs) for breast cancer by disability status using indirect standardization, with national female population rates as the reference. Data were stratified by disability status, 10-year age groups (30–39, 40–49, …, ≥80), and calendar year (2003–2019). Exact 95 % confidence intervals (CIs) were calculated assuming a Poisson distribution (Ulm, 1990). Mortality-to-incidence (M/I) ratios were calculated as adjusted mortality rates divided by adjusted incidence rates to assess potential inequalities in cancer management (Choi et al., 2017; Hebert et al., 2009; Wagner et al., 2012). Between-group comparisons of SIRs, SMRs, and M/I ratios were conducted using the method of Morris and Gardner (1988). Time-to-event analyses were performed using Cox proportional hazards models to estimate hazard ratios (HRs) for breast cancer incidence, breast cancer-specific mortality (regardless of prior diagnosis), and all-cause mortality. To preserve temporality and avoid reverse causality, we excluded 974 women diagnosed with breast cancer before disability registration (Fig. 1). Due to non-proportional hazards, we applied piecewise Cox models across three follow-up intervals (0–<60, 60–<120, and ≥120 months), incorporating time-dependent interactions with disability status. Models were adjusted for all covariates listed above. Given the strong association between age and breast cancer outcomes, age (in 10-year groups) was adjusted for with underlying stratification, allowing the baseline hazard to vary by group. Follow-up began on January 1, 2003, and continued until the event of interest (breast cancer diagnosis or death) or December 31, 2019, whichever came first. Censoring occurred at death or at the end of follow-up. Follow-up time was measured in months.

All analyses were performed using SAS Enterprise Guide 7.1 (SAS Institute Inc., Cary, NC, USA). Additional details on statistical procedures and variable definitions are provided in Supplementary Methods S1.

### 2.6 Ethics statement

This study was approved by the Institutional Review Board of the National Cancer Center, Korea (NCC2022–0180). Informed consent was waived due to the use of de-identified secondary data.

## 3 Results

### 3.1 Study participants

Of the 2,872,871 cancer-free women at baseline in 2002, 296,689 (10.3 %) developed disabilities during follow-up, while 2,576,182 (89.7 %) did not. Women with disabilities were older (mean age: 58.4 vs 49.6 years), more likely to reside in rural areas, had lower income levels, higher rates of smoking and overweight status, lower levels of physical activity, reduced breast cancer screening uptake, and greater comorbidity burdens. All differences were statistically significant (Table 1).

**Table 1:** General characteristics of women aged ≥30 years with and without disabilities in South Korea, 2002–2003.

		Total		Women with disabilities		Women without disabilities		*P*-value ^a^
		n	(%)	n	(%)	n	(%)	
Total		2,872,871	(100.0)	296,689	(100.0)	2,576,182	(100.0)	
Age (Mean ± SD)		50.5 ± 11.8		58.4 ± 10.1		49.6 ± 11.6		<0.0001
Age group	30–39	490,175	(17.1)	9608	(3.2)	480,567	(18.7)	<0.0001
	40–49	1,008,647	(35.1)	52,704	(17.8)	955,943	(37.1)	
	50–59	669,82 7	(23.3)	84,853	(28.6)	584,974	(22.7)	
	60–69	510,241	(17.8)	111,731	(37.7)	398,510	(15.5)	
	70–79	170,446	(5.9)	34,977	(11.8)	135,469	(5.3)	
	≥ 80	23,535	(0.8)	2816	(0.9)	20,719	(0.8)	
Income level	I (Lowest)	537,671	(19.0)	56,267	(19.2)	481,404	(18.9)	<0.0001
	II	450,038	(15.9)	51,451	(17.6)	398,587	(15.7)	
	III	552,085	(19.5)	59,358	(20.3)	492,727	(19.4)	
	IV	577,801	(20.4)	55,979	(19.1)	521,822	(20.5)	
	V (Highest)	719,812	(25.4)	69,709	(23.8)	650,103	(25.6)	
Residential area	Metropolitan	1,564,763	(54.6)	134,117	(45.3)	1,430,646	(55.7)	<0.0001
	City	821,999	(28.7)	90,580	(30.6)	731,419	(28.5)	
	Rural	479,391	(16.7)	71,190	(24.1)	408,201	(15.9)	
Smoking status	Current smoker	78,021	(2.8)	9723	(3.4)	68,298	(2.7)	<0.0001
	Non-smoker	2,702,642	(97.2)	277,382	(96.6)	2,425,260	(97.3)	
Alcohol consumption	≥ 1 per week	209,102	(7.4)	16,811	(5.8)	192,291	(7.6)	<0.0001
	Rarely	2,601,845	(92.6)	272,233	(94.2)	2,329,612	(92.4)	
Physical activity	None	1,902,001	(68.0)	209,787	(72.8)	1,692,214	(67.5)	<0.0001
	≥ 1 per week	893,352	(32.0)	78,421	(27.2)	814,931	(32.5)	
Overweight	Yes	900,151	(31.4)	131,939	(44.5)	768,212	(29.9)	<0.0001
(BMI ≥ 25 kg/m^2^)	No	1,969,791	(68.6)	164,385	(55.5)	1,805,406	(70.2)	
CCI	0	1,435,514	(50.0)	104,294	(35.2)	1,331,220	(51.7)	<0.0001
	1–2	1,216,340	(42.3)	145,645	(49.1)	1,070,695	(41.6)	
	≥ 3	221,017	(7.7)	46,750	(15.8)	174,267	(6.8)	
Breast cancer Screening (≥ 1)	Ever	2,653,736	(92.4)	266,179	(89.6)	2,387,557	(92.5)	<0.0001
(2002–2019)^b^	Never	219,135	(7.6)	30,510	(10.4)	188,625	(7.5)	
Breast cancer diagnosis	Ever	49,846	(1.7)	3579	(1.2)	46,267	(1.8)	<0.0001
(2003–2019)^b^	Never	2,823,025	(98.3)	293,110	(98.8)	2,529,915	(98.2)	
Age at death (Mean ± SD)^b^		77.0 ± 11.4		77.3 ± 9.7		76.9 ± 11.8		<0.0001
Death (2003–2019)^b^	Death	285,017	(9.9)	61,927	(20.9)	223,090	(8.7)	<0.0001
	Survival	2,587,854	(90.1)	234,762	(79.1)	2,353,092	(91.3)	
Causes of death	Breast cancer death	4244	(0.2)	465	(0.2)	3779	(0.2)	<0.0001
(2003–2019) ^c^	Other death	280,773	(9.8)	61,462	(20.7)	219,311	(8.5)	
	Survival	2,587,854	(90.1)	234,762	(79.1)	2,353,092	(91.3)	

**Abbreviations:** SD, standard deviation; CCI, Charlson Comorbidity Index;

All variables are from baseline (2002–2003) unless otherwise noted.

^a^ Student's *t*-test was performed for normally distributed continuous variables, while the Mann-Whitney test was used for non-normally distributed variables. The chi-square test was used to compare categorical variables between women with and without disabilities.

^b^ Breast cancer screening was assessed between 2002 and 2019; breast cancer diagnosis, age at death, death, and causes of death were assessed during the follow-up period (2003–2019).

### 3.2 Standardized incidence and mortality ratios

We observed 4,718,046 person-years for women with disabilities and 42,380,085 person-years for women without, with mean follow-up durations of 16.4 and 15.8 years, respectively. Between 2003 and 2019, 3579 women with disabilities and 46,267 without developed breast cancer, resulting in 465 and 3779 breast cancer-specific deaths, respectively.

**Fig. 2:**
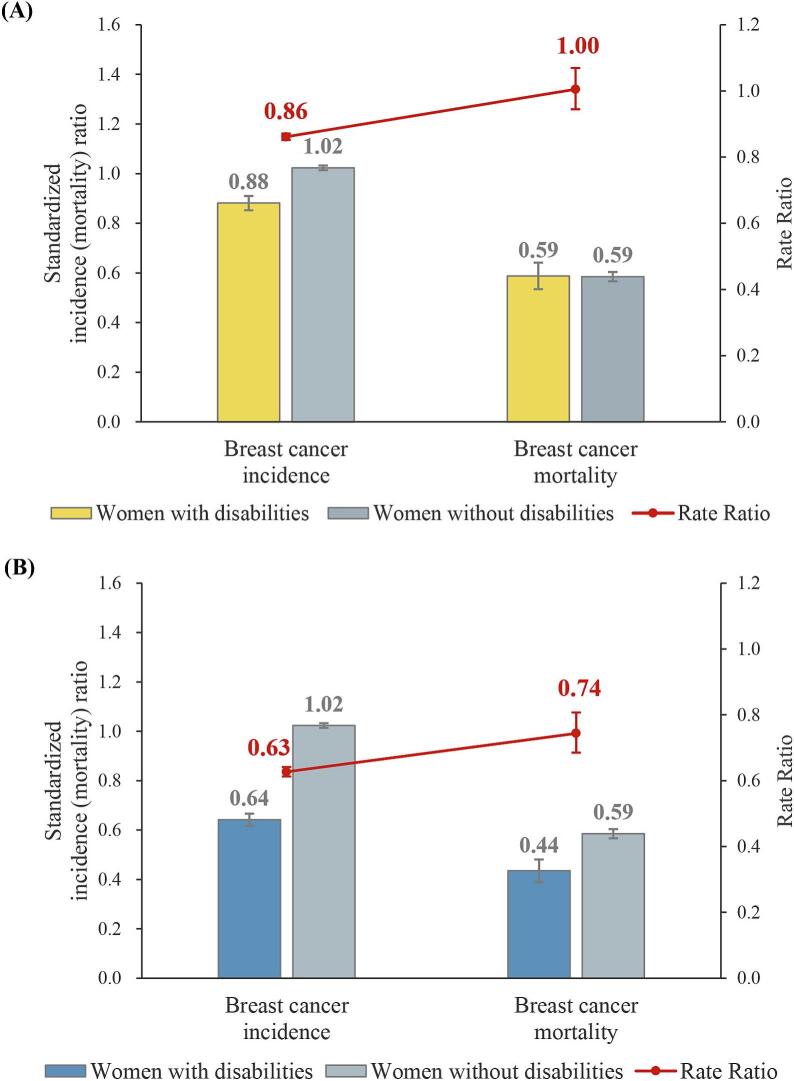
Standardized incidence ratios and standardized mortality ratios of breast cancer, and rate ratios by disability status, Korea, 2003–2019. (A) Standardized incidence and mortality of breast cancer, (B) Breast cancer incidence after disability registration and breast cancer mortality. **Notes:** SIR = standardized incidence ratio; SMR = standardized mortality ratio.

Compared with the general population, women with disabilities had 12 % lower breast cancer incidence and 41 % lower breast cancer-specific mortality (SIR 0.88 [95 % CI 0.85, 0.91]; SMR 0.59 [95 % CI 0.53, 0.64]) (Fig. 2A, Supplementary Tables S1 and S2). Women without disabilities had a slightly higher incidence (SIR 1.02 [95 % CI 1.01, 1.03]) but significantly lower mortality (SMR 0.59 [95 % CI 0.57, 0.60]). Between-group comparisons showed no difference in breast cancer-specific mortality (ratio of SMRs 1.00 [95 % CI 0.89, 1.13]), but a notable difference in incidence (ratio of SIRs 0.86 [95 % CI 0.83, 0.90]). The M/I ratio was significantly higher in the disability group (1.17 [95 % CI 1.10, 1.24]), suggesting poorer outcomes following diagnosis and a greater risk of breast cancer-specific death once diagnosed (Supplementary Table S2).

Among women diagnosed with breast cancer after disability registration, both incidence and mortality were further reduced (SIR 0.64 [95 % CI, 0.62, 0.67]; SMR 0.44 [95 % CI 0.39, 0.48]); however, the M/I ratio remained elevated (1.19 [95 % CI 1.09, 1.29]) (Fig. 2B, Supplementary Table S2).

### 3.3 Differences in breast cancer outcomes and all-cause mortality by disability status

Among 2,695,095 women with complete data, 45,872 developed breast cancer and 266,328 died, including 3845 from breast cancer specifically. The mean follow-up was 16.2 years for breast cancer incidence and 16.4 years for both breast cancer-specific and all-cause mortality.

**Fig. 3:**
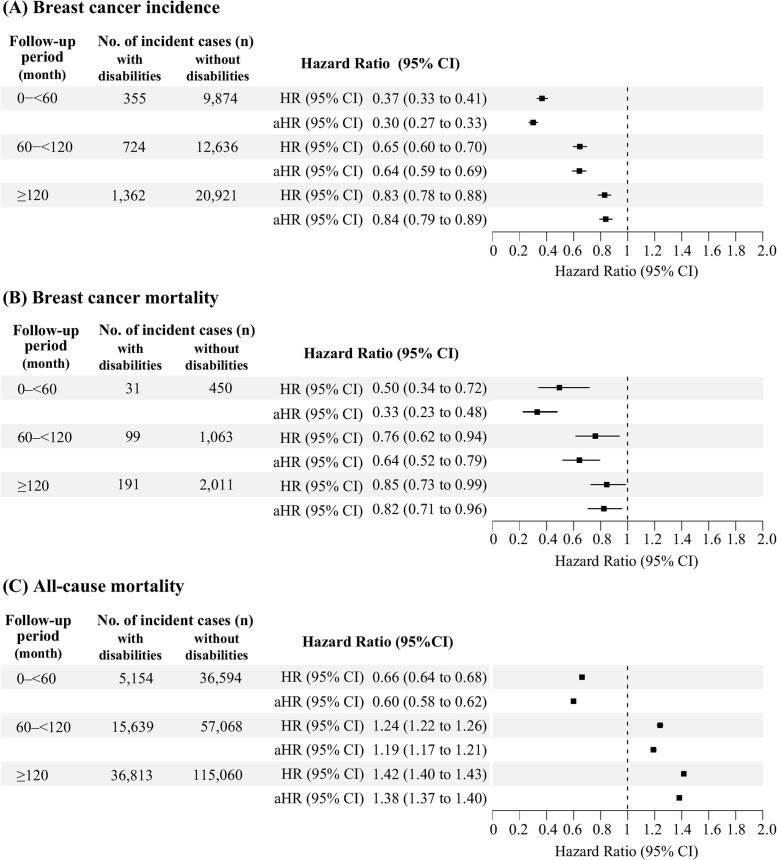
Adjusted hazard ratios for breast cancer incidence, breast cancer-specific mortality, and all-cause mortality among Korean women with and without disabilities within three follow-up intervals. (A) Breast cancer incidence, (B) Breast cancer mortality, (C) All-cause mortality. **Notes:** HR = Hazard ratio; aHR = adjusted hazard ratio, CI = confidence interval. All Cox regression analyses were adjusted for sociodemographic factors (income level, residential area), health behaviors (smoking status, alcohol consumption, physical activity), and clinical factors, including overweight (BMI ≥25 kg/m^2^), CCI (Charlson Comorbidity Index) group, and pre-diagnosis breast cancer screening experience.

Disability was consistently associated with lower breast cancer incidence across all follow-up intervals, with adjusted HRs (aHRs) of 0.30 (95 % CI 0.27, 0.33) for 0–<60 months, 0.64 (95 % CI 0.59, 0.69) for 60–<120 months, and 0.84 (95 % CI 0.79, 0.89) for ≥120 months (Fig. 3A, Supplementary Table S3).

Breast cancer-specific mortality was also lower among women with disabilities, with aHRs of 0.33 (95 % CI 0.23, 0.48), 0.64 (95 % CI 0.52, 0.79), and 0.82 (95 % CI 0.71, 0.96) across the same intervals (Fig. 3B, Supplementary Table S4).

By contrast, all-cause mortality was initially lower (aHR 0.60 [95 % CI 0.58, 0.62]) but increased over time (1.19 [95 % CI 1.17, 1.21] and 1.38 [95 % 1.37, 1.40]), indicating a shift from a protective to adverse effect (Fig. 3C, Supplementary Table S5).

## 4 Discussion

Women with disabilities had lower breast cancer incidence but similar mortality compared with those without, resulting in slightly higher M/I ratios. This suggests potential inequalities in the quality or timeliness of cancer care after diagnosis. The lower incidence was more pronounced among women with preexisting disabilities. Previous studies have reported mixed findings depending on disability type and setting (Iezzoni, 2022; Iezzoni et al., 2021; Parish et al., 2013; Satge et al., 2020). For example, in the U.S., women with complex activity limitations (self-care, social, or work difficulties) had a higher breast cancer risk, while no difference was observed among those with movement disabilities (Iezzoni et al., 2021). In Sweden, older women with intellectual disabilities had lower breast cancer incidence than the general population (Satge et al., 2020). Our findings align with studies reporting lower breast cancer incidence among women with intellectual or physical disabilities.

Disability was associated with persistently lower breast cancer incidence and breast cancer-specific mortality across follow-up intervals. Adjusted HRs showed that incidence and mortality hazards were substantially reduced during the early follow-up period and remained lower over time. In contrast, all-cause mortality, initially lower (suggesting a protective effect of disability), increased significantly over time, eventually surpassing that of women without disabilities.

These divergent trends may reflect competing risks or underlying conditions related to disabilities. Women with disabilities in our study had 3.06 times higher odds of dying from non-breast cancer causes than from breast cancer, relative to women without disabilities (crude OR = 3.06). This highlights the need for further research into competing mortality risks and the broader chronic disease burden in this population.

Factors related to breast cancer screening accessibility were strongly associated with breast cancer incidence, particularly in the early follow-up period (0–<60 months). Never having undergone screening showed the strongest association (aHR 17.41), which diminished to aHR 2.05 at 60–<120 months and 1.15 at ≥120 months. This pattern likely reflects the detection of prevalent cases among screened individuals, with the introduction of the National Cancer Screening Program creating an immediate surge in diagnostic opportunities for pre-existing asymptomatic cancers. The effect subsequently attenuated as these cases were censored from later analyses. Residing in metropolitan areas, where access to healthcare services is typically better, also showed strong associations. Bioclinical factors, such as overweight status, smoking, alcohol consumption, and engaging in regular physical activity (≥1 time per week), were associated with higher breast cancer incidence (Supplementary Table S4).

Notably, sociodemographic, behavioral, and clinical factors—including lower income level, smoking, overweight status, and comorbidities—were consistently associated with higher breast cancer-specific mortality and all-cause mortality. While disability and rural residence were associated with both breast cancer incidence and mortality, lower income was particularly linked to increased breast cancer mortality despite lower incidence. This suggests inequalities in the timeliness or effectiveness of treatment and underscores the critical role of income in shaping prognosis.

Contrary to the patterns observed for breast cancer incidence and mortality, disability and rural residence showed reversed associations with all-cause mortality, and income level had an inverse association with breast cancer incidence but not with breast cancer mortality. These findings may reflect inequalities in cancer detection and management, particularly regarding screening and diagnostic access. All-cause mortality appeared to be primarily driven by poorer overall health, socioeconomic disadvantage, and clinical vulnerability—including disability status, low income, and unfavorable health behaviors.

Persistent inequalities in cancer care access and outcomes among people with disabilities have been documented globally (Iezzoni, 2022). Barriers to cancer care for this population span the continuum of screening, diagnosis, and treatment, including: (i) obstacles to preventive healthcare-seeking behaviors (e.g., disability type and severity, lack of symptom awareness, limited knowledge of services, and competing health priorities) (Edwards et al., 2020; Iezzoni, 2022); (ii) communication difficulties with physicians, including stigmatizing attitudes (Agaronnik et al., 2021; Iezzoni, 2022); (iii) diagnostic overshadowing and systemic bias (Edwards et al., 2020; Seo et al., 2021); and (iv) physical accessibility challenges, such as inaccessible screening equipment and examination tables (Agaronnik et al., 2022).

In Korea, efforts to address these challenges remain limited. The National Cancer Screening Program recommends biennial mammography for women aged ≥40, offered either free of charge or with a 10 % copayment (approximately USD 3.50), depending on income level (Lee et al., 2025; Shin et al., 2020). The lifetime screening rate (2002–2019) was 92.4 %, higher than the previously reported 82.5 % (2004–2019) (Kang et al., 2024). However, in our study population, women with disabilities had slightly reduced access to National Cancer Screening Program breast cancer screening (rate ratio [RR] 0.97, 95 % CI 0.96, 0.97) (Supplementary Fig. S1), consistent with national trends (RR 0.89 in 2015) (Shin et al., 2020). This disparity may be underestimated, as our sample included only voluntary health checkup participants, who were likely more health-conscious than the general population.

The Disability-Friendly General Medical Examination Program, introduced in 2017 under the Act on Guarantee of the Right to Health and Access to Medical Services for Persons with Disabilities, aims to improve access to general medical examinations for individuals with disabilities. However, coverage remains limited: as of 2021, fewer than 100 designated medical institutions were available, and access was restricted to officially registered individuals (National Rehabilitation Center, 2024).

This study had several limitations. First, the study population comprised women who voluntarily participated in the National Health Screening Program, representing about 20.7 % of Korean women. These participants may have been more health-conscious and better resourced than the general population, potentially underestimating both breast cancer incidence and related inequalities. Second, disability status was analyzed without accounting for disability type or severity due to data limitations. Future research should explore these differences to identify target populations for intervention. Third, we relied on the first year of disability registration as a proxy for disability onset, which may not fully capture the true duration of disability exposure. Finally, the temporal relationship between disability onset and cancer diagnosis could not be firmly established, limiting causal inferences.

## 5 Conclusion

Our findings underscore persistent inequalities in breast cancer outcomes among women with disabilities in Korea, despite lower incidence. These inequities appear to stem from delayed screening, later diagnosis, and systemic barriers to cancer care. Addressing these gaps requires policy measures that ensure equitable access throughout the cancer care continuum, including prevention, early detection, treatment, and survivorship. Future research should prioritize the design, implementation, and evaluation of disability-inclusive interventions and examine the role of disability type and severity in shaping cancer outcomes.

## CRediT authorship contribution statement

**Hee-Yeon Kang:** Writing – review & editing, Writing – original draft, Project administration, Methodology, Investigation, Formal analysis, Data curation. **Eunjung Park:** Writing – review & editing, Methodology, Data curation. **Thi Tra Bui:** Writing – review & editing, Methodology. **Byungmi Kim:** Writing – review & editing, Methodology, Funding acquisition. **Jin-Kyoung Oh:** Writing – review & editing, Writing – original draft, Supervision, Project administration, Methodology, Investigation, Funding acquisition, Data curation, Conceptualization.

## Funding

This study was supported by the 10.13039/100008746National Cancer Center, Republic of Korea (grant numbers: NCC-2210862 and NCC-2310652).

## Declaration of competing interest

The authors declare that they have no known competing financial interests or personal relationships that could have appeared to influence the work reported in this paper.

## Data Availability

The data used in this study (NHIS-2022-1-785) were provided by the National Health Insurance Service (NHIS) of Korea with permission from the National Health Insurance Data Review Committee (http://nhiss.nhis.or.kr). Data could not be shared because of the participants' confidentiality. Additional summary-level data without individual data can be provided upon reasonable request, with permission from the NHIS.

## Appendix A Supplementary data

Supplementary material Image 1
