# Supplementary material for: Inequalities in breast cancer incidence and mortality in women with and without disabilities in South Korea: A population-based cohort study
Source: Prev Med Rep. 2025 Sep 12;59:103242. doi: 10.1016/j.pmedr.2025.103242 (PMC12476072; doi:10.1016/j.pmedr.2025.103242)
Supplement: Supplementary file 1 — Supplementary material [file mmc1.docx]

**Supplementary Materials**

**Supplementary Methods S1.** Detailed statistical methods for standardized rate calculations and regression analyses.

**Supplementary Table S1.** Study participants from Korea’s 2002–2003 National Screening Program, incident breast cancer cases, and deaths by age group and disability status, 2003–2019.

**Supplementary Table S2.** Standardized incidence ratio (SIR), standardized mortality ratio (SMR), and standardized rates of breast cancer, and corresponding rate ratios among Korean women with and without disabilities, 2003–2019.

**Supplementary Table S3.** Adjusted hazard ratios and 95% confidence intervals for breast cancer incidence by disability status across follow-up intervals among Korean women, 2003–2019.

**Supplementary Table S4.** Adjusted hazard ratios and 95% confidence intervals for breast cancer-specific mortality by disability status across follow-up intervals among Korean women, 2003–2019.

**Supplementary Table S5.** Adjusted hazard ratios and 95% confidence intervals for all-cause mortality by disability status across follow-up intervals among Korean women, 2003–2019.

**Supplementary Fig. S1.** Age- and 2-year calendar period-standardized national breast cancer screening rates (per 100 person-years) and rate ratio among Korean women with and without disabilities, 2002–2019.

**Supplementary Methods S1.** Detailed statistical methods for standardized rate calculations and regression analyses.

***Methods for the calculation of Standardized Incidence Rates and Standardized Mortality Rates***

We calculated age- and calendar year-adjusted standardized incidence ratios (SIRs) and standardized mortality ratios (SMRs) for breast cancer (BRC) by disability status using indirect standardization. This method was chosen to enable relative comparisons with the general population, given the incomplete coverage of the general health screening program. Age- and calendar year-specific BRC incidence and mortality rates for the general population were obtained from the Korea Central Cancer Registry (KCCR) and Statistics Korea, respectively, and used as reference rates. Given the high completeness of both data sources, near-complete follow-up was assumed to be accurate. Aggregated counts of incident BRC, BRC-specific deaths, and all-cause deaths were stratified by calendar year (2003–2019), age group (10-year intervals to ≥80), and disability status. Person-years were estimated based on aggregated data across these strata, without applying individual-level censoring at the time of diagnosis or death. To account for time at risk, 0.5 person-years were subtracted for each incident case or death from total annual person-years, assuming that cases and deaths occurred, on average, at the midpoint of the year. This adjustment helped avoid overestimation of time at risk. Expected numbers were calculated by multiplying the reference rates by person-years in each stratum. The SIRs and SMRs were defined as the ratios of observed to expected cases or deaths, respectively. Exact 95% confidence intervals (CIs) were estimated assuming a Poisson distribution (Ulm, 1990). Indirectly adjusted incidence rates (AIRs) and adjusted mortality rates (AMRs) were also calculated by multiplying the SIRs (or SMRs) by the reference crude rates. All standardized rates were calculated using the PROC STDRATE procedure in SAS.

Mortality-to-incidence (M/I) ratios were computed as the ratios of AMRs to AIRs within each group to assess potential inequalities in cancer management outcomes (Hebert et al., 2009; Wagner et al., 2012).

Finally, to compare SIRs, SMRs, and M/I ratios between women with and without disabilities, we estimated the ratios of each indicator and their 95% CIs using the method proposed by Morris and Gardner (1988), which is specifically designed for comparing independent standardized ratios.

***Time-to-Event Analyses: Association of Disability Status with Breast Cancer Incidence, Breast Cancer-specific Mortality, and All-cause Mortality***

***Statistical analyses***

Cox proportional hazards models were used to estimate hazard ratios (HRs) for the associations between disability status and the risks of BRC incidence, BRC-specific mortality (regardless of prior BRC diagnosis), and all-cause mortality.

To preserve temporal sequence between exposure and outcome, women with disabilities who were diagnosed with BRC prior to disability registration (n=974) were excluded from the analysis (Fig. S1). We acknowledge that the date of disability registration may not reflect the actual onset of disability, and some individuals may have had pre-existing conditions influencing both disability registration timing and cancer detection patterns.

Participants were followed from January 1, 2003, until the occurrence of the outcome of interest or December 31, 2019, whichever came first. For BRC incidence, participants were censored at death or the end of follow-up if no BRC diagnosis occurred. For BRC-specific mortality, deaths from other causes were treated as censored events. For all-cause mortality, participants who remained alive were censored at the end of follow-up. Follow-up time was measured in months.

The proportional hazards assumption was assessed using log(-log) survival plots and Schoenfeld residuals. Non-proportional hazards were observed for disability status, age group, Charlson Comorbidity Index (CCI), and BRC screening history. To address this, we applied piecewise Cox models with predefined follow-up intervals (0–<5 years [0–<60 months], 5–10 years [60–<120 months], and ≥10 years [≥120 months]) (Royston & Parmar, 2002).

In the Cox proportional hazards models, given its strong association with BRC outcomes, age (in 10-year groups) was adjusted for using underlying stratification, which allowed the baseline hazard to vary by age group. Age group, similar to disability status, was the strongest driver of non-proportional hazards—its effect on BRC risk and mortality changed substantially over time and violated the proportional hazards assumption more severely than any other covariate.

Breslow, N.E., Day, N.E., 1987. Statistical methods in cancer research. Volume II--The design and analysis of cohort studies. IARC Sci Publ:1-406.

Charlson, M.E., Pompei, P., Ales, K.L., MacKenzie, C.R., 1987. A new method of classifying prognostic comorbidity in longitudinal studies: development and validation. J Chronic Dis 40:373-83. https://doi.org/10.1016/0021-9681(87)90171-8

Hebert, J.R., Daguise, V.G., Hurley, D.M., Wilkerson, R.C., Mosley, C.M., Adams, S.A., Puett, R., Burch, J.B., Steck, S.E., et al., 2009. Mapping cancer mortality-to-incidence ratios to illustrate racial and sex disparities in a high-risk population. Cancer 115:2539-52. <https://doi.org/10.1002/cncr.24270>.Morris, J.A., Gardner, M.J., 1988. Calculating confidence intervals for relative risks (odds ratios) and standardised ratios and rates. Br Med J (Clin Res Ed) 296:1313-6.

Quan, H., Sundararajan, V., Halfon, P., Fong, A., Burnand, B., Luthi, J.C., Saunders, L.D., Beck, C.A., Feasby, T.E., et al., 2005. Coding algorithms for defining comorbidities in ICD-9-CM and ICD-10 administrative data. Med Care 43:1130-9. https://doi.org/10.1097/01.mlr.0000182534.19832.83

Royston, P., Parmar, M.K., 2002. Flexible parametric proportional-hazards and proportional-odds models for censored survival data, with application to prognostic modelling and estimation of treatment effects. Stat Med 21:2175-97.Ulm, K., 1990. A simple method to calculate the confidence interval of a standardized mortality ratio (SMR). Am J Epidemiol 131:373-5. https://doi.org/10.1093/oxfordjournals.aje.a115507.

Wagner, S.E., Hurley, D.M., Hebert, J.R., McNamara, C., Bayakly, A.R., Vena, J.E., 2012. Cancer mortality-to-incidence ratios in Georgia: describing racial cancer disparities and potential geographic determinants. Cancer 118:4032-45. https://doi.org/10.1002/cncr.26728.

Supplementary Table S1. Study participants from Korea’s 2002–2003 National Health Screening Program, incident breast cancer cases, and deaths by age group and disability status, 2003–2019.

| **Age-group** | **Total**  **population** |  | **Women with disabilities** | | | | | | | | | | |  | **Women without disabilities** | | | | | | | | |
| --- | --- | --- | --- | --- | --- | --- | --- | --- | --- | --- | --- | --- | --- | --- | --- | --- | --- | --- | --- | --- | --- | --- | --- |
|  |  |  | **Baseline** | | **Person-years** | **Incident  cases** | | **Incident cases after disability** | | **Deaths** | | **Breast cancer**  **deaths** | |  | **Baseline** | | **Person- years** | **Incident  cases** | | **Deaths** | | **Breast cancer**  **deaths** | |
|  | **n** |  | **n** | **(%)** | **n** | **n** | **(%)** | **n** | **(%)** | **n** | **(%)** | **n** | **(%)** |  | **n** | **(%)** | **n** | **n** | **(%)** | **n** | **(%)** | **n** | **(%)** |
| Total | 2,872,871 |  | 296,689 | (100) | 4,718,046 | 3,579 | (100) | 2,605 | (100) | 61,927 | (100) | 465 | (100) |  | 2,576,182 | (100) | 42,380,085 | 46,267 | (100) | 223,090 | (100) | 3,779 | (100) |
| 30-39 | 490,175 |  | 9,608 | (3.2) | 30,708 | 24 | (0.7) | 8 | (0.3) | 41 | (0.1) | 2 | (0.4) |  | 480,567 | (18.7) | 2,009,827 | 960 | (2.1) | 605 | (0.3) | 50 | (1.3) |
| 40-49 | 1,008,647 |  | 52,704 | (17.8) | 304,162 | 376 | (10.5) | 211 | (8.1) | 573 | (0.9) | 30 | (6.5) |  | 955,943 | (37.1) | 9,179,113 | 12,454 | (26.9) | 5,558 | (2.5) | 475 | (12.6) |
| 50-59 | 669,827 |  | 84,853 | (28.6) | 890,897 | 940 | (26.3) | 658 | (25.3) | 2,775 | (4.5) | 128 | (27.5) |  | 584,974 | (22.7) | 13,512,703 | 17,449 | (37.7) | 16,018 | (7.2) | 1,328 | (35.1) |
| 60-69 | 510,241 |  | 111,731 | (37.7) | 1,499,909 | 1,146 | (32.0) | 853 | (32.7) | 8,189 | (13.2) | 118 | (25.4) |  | 398,510 | (15.5) | 9,971,441 | 10,518 | (22.7) | 28,961 | (13.0) | 976 | (25.8) |
| 70-79 | 170,446 |  | 34,977 | (11.8) | 1,456,205 | 868 | (24.3) | 684 | (26.3) | 22,366 | (36.1) | 114 | (24.5) |  | 135,469 | (5.3) | 5,709,350 | 4,027 | (8.7) | 63,531 | (28.5) | 604 | (16.0) |
| 80+ | 23,535 |  | 2,816 | (1.0) | 536,165 | 225 | (6.3) | 191 | (7.3) | 27,983 | (45.2) | 73 | (15.7) |  | 20,719 | (0.8) | 1,997,651 | 859 | (1.9) | 108,417 | (48.6) | 346 | (9.2) |

Supplementary Table S2. Standardized incidence ratio (SIR), standardized mortality ratio (SMR), standardized rates of breast cancer, and corresponding rate ratios among Korean women with and without disabilities, 2003–2019.

|  |  | **Women with disabilities (A)** | | | |  | **Women without disabilities (B)** | | | |  | **Between-group ratio (A/B)** | |
| --- | --- | --- | --- | --- | --- | --- | --- | --- | --- | --- | --- | --- | --- |
|  |  | **n** | **SIR (SMR)**  **(95% CI)** | **Adjusted rate ^c^**  **(95% CI)** | **M/I ratio**  **(95% CI)** |  | **n** | **SIR (SMR)**  **(95% CI)** | **Adjusted rate ^c^**  **(95% CI)** | **M/I ratio**  **(95% CI)** |  | **SIR (SMR)**  **(95% CI)** | **M/I ratio**  **(95% CI)** |
| Incidence and mortality of breast cancer (Ⅰ) ^a^ | | |  |  |  |  |  |  |  |  |  |  |  |
| Total | | 296,689 |  |  |  |  | 2,576,182 |  |  |  |  |  |  |
|  | Incident breast cancer cases | 3,579 | 0.88  (0.85, 0.91) | 93.08  (90.03, 96.13) | 0.09  (0.08, 0.09) |  | 46,267 | 1.02  (1.01, 1.03) | 108.10  (107.10, 109.00) | 0.08  (0.07, 0.08) |  | 0.86  (0.83, 0.90) | 1.17  (1.10, 1.24) |
|  | Breast cancer deaths | 465 | 0.59  (0.53, 0.64) | 8.20  (7.45, 8.95) |  |  | 3,779 | 0.59  (0.57, 0.60) | 8.16  (7.90, 8.42) |  |  | 1.00  (0.89, 1.13) |  |
|  | All-cause deaths | 61,927 | 0.93  (0.92, 0.93) | 765.10  (759.10, 771.20) |  |  | 223,090 | 0.73  (0.73, 0.74) | 606.10  (603.50, 608.60) |  |  | 1.26  (1.25, 1.28) |  |
| Incidence and mortality of breast cancer (Ⅱ) ^b^ | | |  |  |  |  |  |  |  |  |  |  |  |
| Total | | 295,715 |  |  |  |  | 2,576,182 |  |  |  |  |  |  |
|  | Incident breast cancer cases | 2,605 | 0.64  (0.62, 0.67) | 68.08  (65.47, 70.70) | 0.09  (0.08, 0.10) |  | 46,267 | 1.02  (1.01, 1.03) | 109.10  (108.10, 110.10) | 0.08  (0.07, 0.08) |  | 0.63  (0.61, 0.64) | 1.19  (1.09, 1.29) |
|  | Breast cancer deaths | 344 | 0.44  (0.39, 0.48) | 6.07  (5.43, 6.71) |  |  | 3,779 | 0.59  (0.57, 0.60) | 8.16  (7.90, 8.42) |  |  | 0.74  (0.69, 0.81) |  |

**Abbreviations**: SIR, standardized incidence ratio; SMR, standardized mortality ratio; CI, confidence interval; M/I ratio, mortality-to-incidence ratio.

^a^ Incidence and mortality (Ⅰ): Incidence and mortality during the follow-up period (2003–2019).

^b^ Incidence and mortality (Ⅱ): Incidence and mortality among women with disabilities (who had never been diagnosed with BRC before disability registration) and among women without disabilities.

^c^ Adjusted rate: Age- and calendar year-adjusted incidence (or mortality) rate using indirect standardization

Supplementary Table S3. Adjusted hazard ratios and 95% confidence intervals for breast cancer incidence by disability status across follow-up intervals among Korean women, 2003–2019.

|  | | **Follow-up period ^a^** | | | | | | | | | | | |
| --- | --- | --- | --- | --- | --- | --- | --- | --- | --- | --- | --- | --- | --- |
|  |  | **0–<60 months**  **(n = 2,695,095)** | | | | **60–<120 months**  **(n = 2,643,525)** | | | | **≥120 months**  **(n = 2,558,717)** | | | |
| **Covariates** | | **HR (95% CI) ^b^** | | **aHR (95% CI) ^c^** | | **HR (95% CI) ^b^** | | **aHR (95% CI) ^c^** | | **HR (95% CI) ^b^** | | **aHR (95% CI) ^c^** | |
| **No. of events** | | **10,229** | | | | **13,360** | | | | **22,283** | | | |
| Disability status ^d^ | |  |  |  |  |  |  |  |  |  |  |  |  |
|  | Yes vs no | 0.37 | (0.33, 0.41) | 0.30 | (0.27, 0.33) | 0.65 | (0.60, 0.70) | 0.64 | (0.59, 0.69) | 0.83 | (0.78, 0.88) | 0.84 | (0.79, 0.89) |
| Residential area | |  |  |  |  |  |  |  |  |  |  |  |  |
|  | City vs metropolitan |  |  | 0.88 | (0.86, 0.90) |  |  | 0.88 | (0.86, 0.90) |  |  | 0.88 | (0.86, 0.90) |
|  | Rural vs metropolitan |  |  | 0.71 | (0.69, 0.73) |  |  | 0.71 | (0.69, 0.73) |  |  | 0.71 | (0.69, 0.73) |
| Income level | |  |  |  |  |  |  |  |  |  |  |  |  |
|  | I (Lowest) vs Ⅴ (Highest) |  |  | 0.82 | (0.80, 0.84) |  |  | 0.82 | (0.80, 0.84) |  |  | 0.82 | (0.80, 0.84) |
|  | Ⅱ vs Ⅴ (Highest) |  |  | 0.87 | (0.84, 0.89) |  |  | 0.87 | (0.84, 0.89) |  |  | 0.87 | (0.84, 0.89) |
|  | Ⅲ vs Ⅴ (Highest) |  |  | 0.91 | (0.88, 0.93) |  |  | 0.91 | (0.88, 0.93) |  |  | 0.91 | (0.88, 0.93) |
|  | Ⅳ vs Ⅴ (Highest) |  |  | 0.94 | (0.92, 0.97) |  |  | 0.94 | (0.92, 0.97) |  |  | 0.94 | (0.92, 0.97) |
| Smoking status | |  |  |  |  |  |  |  |  |  |  |  |  |
|  | Current smoker vs non-smoker |  |  | 1.06 | (1.00, 1.13) |  |  | 1.06 | (1.00, 1.13) |  |  | 1.06 | (1.00, 1.13) |
| Alcohol consumption | |  |  |  |  |  |  |  |  |  |  |  |  |
|  | ≥1 per week vs rarely drinking |  |  | 1.04 | (1.00, 1.07) |  |  | 1.04 | (1.00, 1.07) |  |  | 1.04 | (1.00, 1.07) |
| Physical activity | |  |  |  |  |  |  |  |  |  |  |  |  |
|  | None vs ≥1 per week |  |  | 0.92 | (0.90, 0.94) |  |  | 0.92 | (0.90, 0.94) |  |  | 0.92 | (0.90, 0.94) |
| Overweight (BMI ≥ 25kg/m^2^) | |  |  |  |  |  |  |  |  |  |  |  |  |
|  | Yes vs no |  |  | 1.14 | (1.11, 1.16) |  |  | 1.14 | (1.11, 1.16) |  |  | 1.14 | (1.11, 1.16) |
| CCI ^d^ | |  |  |  |  |  |  |  |  |  |  |  |  |
|  | 1–2 vs 0 |  |  | 1.24 | (1.19, 1.30) |  |  | 1.04 | (1.01, 1.08) |  |  | 1.01 | (0.98, 1.04) |
|  | ≥3 vs 0 |  |  | 3.66 | (3.45, 3.88) |  |  | 1.05 | (0.97, 1.13) |  |  | 1.04 | (0.98, 1.11) |
| BRC screening (2002–2019) ^d^ | |  |  |  |  |  |  |  |  |  |  |  |  |
|  | Never vs ever |  |  | 17.41 | (16.71, 18.14) |  |  | 4.05 | (3.86, 4.25) |  |  | 1.15 | (1.09, 1.22) |

**Abbreviations:** HR, hazard ratio; aHR, adjusted hazard ratio; CI, confidence interval; CCI, Charlson Comorbidity Index.

^a^ In the piecewise model, breast cancer incidence was defined as events that occurred within about 0–<5 years (0–<60 months), 5–<10 years (60–<120 months), and ≥10 years (≥120 months) of follow-up.

^b^ Unadjusted models stratified by age groups

^c^ Adjusted models were conducted, adjusting for age group, residential area, income quintile, smoking status, alcohol consumption, physical activity, overweight, CCI score (2002–2003), and breast cancer screening experience during 2002–2019.

^d^ Variables that violated the proportional hazards assumption (disability status, CCI, and breast cancer screening experience) were modeled with time-period-specific hazard ratios using piecewise Cox regression.

Supplementary Table S4. Adjusted hazard ratios and 95% confidence intervals for breast cancer-specific mortality by disability status across follow-up intervals among Korean women, 2003–2019.

|  | | **Follow-up period ^a^** | | | | | | | | | | | |
| --- | --- | --- | --- | --- | --- | --- | --- | --- | --- | --- | --- | --- | --- |
|  |  | **0–<60 months**  **(n = 2,695,095)** | | | | **60–<120 months**  **(n = 2,653,347)** | | | | **≥120 months**  **(n = 2,580,640)** | | | |
| **Covariates** | | **HR (95% CI) ^b^** | | **aHR (95% CI) ^c^** | | **HR (95% CI) ^b^** | | **aHR (95% CI) ^c^** | | **HR (95% CI) ^b^** | | **aHR (95% CI) ^c^** | |
| **No. of events** | | **481** | | | | **1,162** | | | | **2,202** | | | |
| Disability status ^d^ | |  |  |  |  |  |  |  |  |  |  |  |  |
|  | Yes vs No | 0.50 | (0.34, 0.72) | 0.33 | (0.23, 0.48) | 0.76 | (0.62, 0.94) | 0.64 | (0.52, 0.79) | 0.85 | (0.73, 0.99) | 0.82 | (0.71, 0.96) |
| Residential area | |  |  |  |  |  |  |  |  |  |  |  |  |
|  | City vs metropolitan |  |  | 0.93 | (0.86, 1.00) |  |  | 0.93 | (0.86, 1.00) |  |  | 0.93 | (0.86, 1.00) |
|  | Rural vs metropolitan |  |  | 0.84 | (0.76, 0.93) |  |  | 0.84 | (0.76, 0.93) |  |  | 0.84 | (0.76, 0.93) |
| Income level | |  |  |  |  |  |  |  |  |  |  |  |  |
|  | I (Lowest) vs Ⅴ (Highest) |  |  | 1.14 | (1.03, 1.25) |  |  | 1.14 | (1.03, 1.25) |  |  | 1.14 | (1.03, 1.25) |
|  | Ⅱ vs Ⅴ (Highest) |  |  | 1.28 | (1.16, 1.42) |  |  | 1.28 | (1.16, 1.42) |  |  | 1.28 | (1.16, 1.42) |
|  | Ⅲ vs Ⅴ (Highest) |  |  | 1.22 | (1.11, 1.34) |  |  | 1.22 | (1.11, 1.34) |  |  | 1.22 | (1.11, 1.34) |
|  | Ⅳ vs Ⅴ (Highest) |  |  | 1.06 | (0.96, 1.16) |  |  | 1.06 | (0.96, 1.16) |  |  | 1.06 | (0.96, 1.16) |
| Smoking status | |  |  |  |  |  |  |  |  |  |  |  |  |
|  | Current smoker vs non-smoker |  |  | 1.11 | (0.92, 1.33) |  |  | 1.11 | (0.92, 1.33) |  |  | 1.11 | (0.92, 1.33) |
| Alcohol consumption | |  |  |  |  |  |  |  |  |  |  |  |  |
|  | ≥1 per week vs rarely drinking |  |  | 0.98 | (0.87, 1.11) |  |  | 0.98 | (0.87, 1.11) |  |  | 0.98 | (0.87, 1.11) |
| Physical activity | |  |  |  |  |  |  |  |  |  |  |  |  |
|  | None vs ≥1 per week |  |  | 0.94 | (0.87, 1.00) |  |  | 0.94 | (0.87, 1.00) |  |  | 0.94 | (0.87, 1.00) |
| Overweight (BMI ≥ 25kg/m^2^) | |  |  |  |  |  |  |  |  |  |  |  |  |
|  | Yes vs No |  |  | 1.21 | (1.13, 1.29) |  |  | 1.21 | (1.13, 1.29) |  |  | 1.21 | (1.13, 1.29) |
| CCI ^d^ | |  |  |  |  |  |  |  |  |  |  |  |  |
|  | 1–2 vs 0 |  |  | 1.42 | (1.12, 1.80) |  |  | 1.08 | (0.95, 1.23) |  |  | 0.96 | (0.88, 1.05) |
|  | ≥3 vs 0 |  |  | 10.28 | (8.16, 12.97) |  |  | 3.25 | (2.75, 3.83) |  |  | 1.34 | (1.15, 1.57) |
| BRC screening (2002–2019) ^d^ | |  |  |  |  |  |  |  |  |  |  |  |  |
|  | Never vs ever |  |  | 36.96 | (30.34, 45.01) |  |  | 16.44 | (14.52, 18.62) |  |  | 5.93 | (5.35, 6.59) |

**Abbreviations:** HR, hazard ratio; aHR, adjusted hazard ratio; CI, confidence interval; CCI, Charlson Comorbidity Index.

^a^ In the piecewise model, breast cancer-specific mortality was defined as events that occurred within 0–< 5 years (0–<60 months), 5–<10 years (60–<120 months), and ≥10 years (≥120 months) of follow-up.

^b^ Unadjusted models stratified by age groups

^c^ Adjusted models were conducted, adjusting for age group, residential area, income quintile, smoking status, alcohol consumption, physical activity, overweight, CCI score (2002–2003), and breast cancer screening experience during 2002–2019.

^d^ Variables that violated the proportional hazards assumption (disability status, CCI, and breast cancer screening experience) were modeled with time-period-specific hazard ratios using piecewise Cox regression.

Supplementary Table S5. Adjusted hazard ratios and 95% confidence intervals for all-cause mortality by disability status across follow-up intervals among Korean women, 2003–2019.

|  | | **Follow-up period ^a^** | | | | | | | | | | | |
| --- | --- | --- | --- | --- | --- | --- | --- | --- | --- | --- | --- | --- | --- |
|  |  | **0–<60 months**  **(n = 2,695,095)** | | | | **60–<120 months**  **(n = 2,653,347)** | | | | **≥120 months**  **(n = 2,580,640)** | | | |
| **Covariates** | | **HR (95% CI) ^b^** | | **aHR (95% CI) ^c^** | | **HR (95% CI) ^b^** | | **aHR (95% CI) ^c^** | | **HR (95% CI) ^b^** | | **aHR (95% CI) ^c^** | |
| **No. of events** | | **41,748** | | | | **72,707** | | | | **151,873** | | | |
| Disability status ^d^ | |  |  |  |  |  |  |  |  |  |  |  |  |
|  | Yes vs No | 0.66 | (0.42, 0.68) | 0.60 | (0.58, 0.62) | 1.24 | (1.22, 1.26) | 1.19 | (1.17, 1.21) | 1.42 | (1.40, 1.43) | 1.38 | (1.37, 1.40) |
| Residential area | |  |  |  |  |  |  |  |  |  |  |  |  |
|  | City vs metropolitan |  |  | 1.13 | (1.12, 1.14) |  |  | 1.13 | (1.12, 1.14) |  |  | 1.13 | (1.12, 1.14) |
|  | Rural vs metropolitan |  |  | 1.30 | (1.29, 1.31) |  |  | 1.30 | (1.29, 1.31) |  |  | 1.30 | (1.29, 1.31) |
| Income level | |  |  |  |  |  |  |  |  |  |  |  |  |
|  | I (Lowest) vs Ⅴ (Highest) |  |  | 1.34 | (1.33, 1.36) |  |  | 1.34 | (1.33, 1.36) |  |  | 1.34 | (1.33, 1.36) |
|  | Ⅱ vs Ⅴ (Highest) |  |  | 1.33 | (1.31, 1.34) |  |  | 1.33 | (1.31, 1.34) |  |  | 1.33 | (1.31, 1.34) |
|  | Ⅲ vs Ⅴ (Highest) |  |  | 1.19 | (1.18, 1.21) |  |  | 1.19 | (1.18, 1.21) |  |  | 1.19 | (1.18, 1.21) |
|  | Ⅳ vs Ⅴ (Highest) |  |  | 1.09 | (1.08, 1.10) |  |  | 1.09 | (1.08, 1.10) |  |  | 1.09 | (1.08, 1.10) |
| Smoking status | |  |  |  |  |  |  |  |  |  |  |  |  |
|  | Current smoker vs non-smoker |  |  | 1.56 | (1.54, 1.58) |  |  | 1.56 | (1.54, 1.58) |  |  | 1.56 | (1.54, 1.58) |
| Alcohol consumption | |  |  |  |  |  |  |  |  |  |  |  |  |
|  | ≥1 per week vs rarely drinking |  |  | 0.94 | (0.92, 0.95) |  |  | 0.94 | (0.92, 0.95) |  |  | 0.94 | (0.92, 0.95) |
| Physical activity | |  |  |  |  |  |  |  |  |  |  |  |  |
|  | None vs ≥1 per week |  |  | 1.14 | (1.13, 1.15) |  |  | 1.14 | (1.13, 1.15) |  |  | 1.14 | (1.13, 1.15) |
| Overweight (BMI ≥ 25kg/m^2^) | |  |  |  |  |  |  |  |  |  |  |  |  |
|  | Yes vs No |  |  | 0.95 | (0.94, 0.96) |  |  | 0.95 | (0.94, 0.96) |  |  | 0.95 | (0.94, 0.96) |
| CCI ^d^ | |  |  |  |  |  |  |  |  |  |  |  |  |
|  | 1–2 vs 0 |  |  | 1.34 | (1.31, 1.37) |  |  | 1.18 | (1.16, 1.20) |  |  | 1.15 | (1.13, 1.16) |
|  | ≥3 vs 0 |  |  | 2.72 | (2.65, 2.79) |  |  | 1.76 | (1.72, 1.80) |  |  | 1.59 | (1.56, 1.61) |
| BRC screening (2002–2019) ^d^ | |  |  |  |  |  |  |  |  |  |  |  |  |
|  | Never vs ever |  |  | 15.72 | (15.35, 16.09) |  |  | 4.14 | (4.07, 4.21) |  |  | 2.24 | (2.21, 2.27) |

**Abbreviations:** HR, hazard ratio; aHR, adjusted hazard ratio; CI, confidence interval; CCI, Charlson Comorbidity Index.

^a^ In the piecewise model, all-cause mortality was defined as events that occurred within 0–< 5 years (0–<60 months), 5–<10 years (60–<120 months), and ≥10 years (≥120 months) of follow-up.

^b^ Unadjusted models stratified by age groups

^c^ Adjusted models were conducted, adjusting for age group, residential area, income quintile, smoking status, alcohol consumption, physical activity, overweight, CCI score (2002–2003), and breast cancer screening experience during 2002–2019.

^d^ Variables that violated the proportional hazards assumption (disability status, CCI, and breast cancer screening experience) were modeled with time-period-specific hazard ratios using piecewise Cox regression.


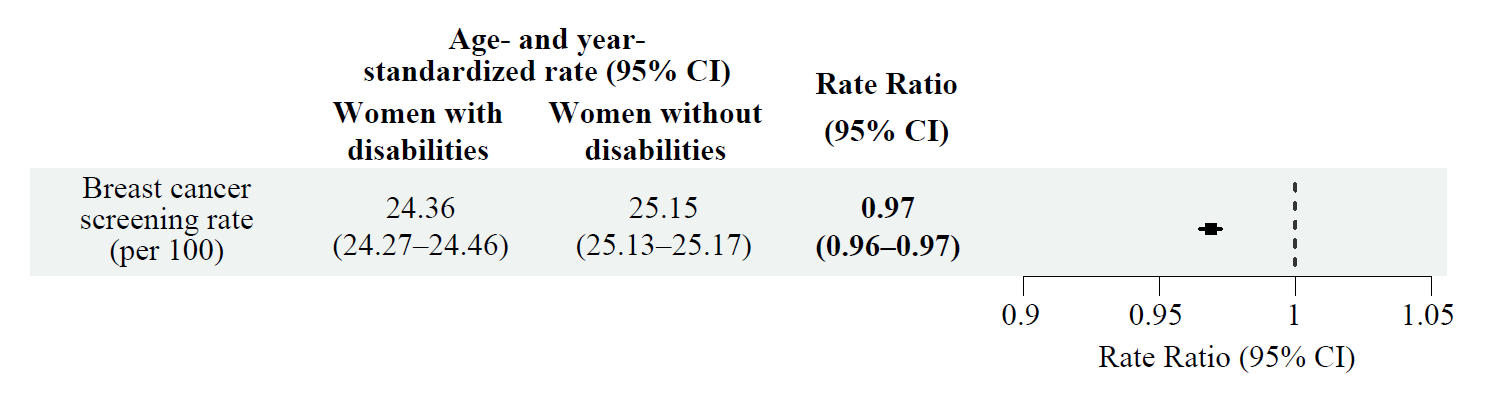
**Supplementary Fig. S1.** Age- and 2-year calendar period-standardized national breast cancer screening rates (per 100 person-years) and rate ratio among Korean women with and without disabilities, 2002–2019.

Note: CI = confidence interval.
